# Oxidative Stress Alters the Profile of Transcription Factors Related to Early Development on *In Vitro* Produced Embryos

**DOI:** 10.1155/2017/1502489

**Published:** 2017-10-25

**Authors:** Roberta Ferreira Leite, Kelly Annes, Jessica Ispada, Camila Bruna de Lima, Érika Cristina dos Santos, Patricia Kubo Fontes, Marcelo Fábio Gouveia Nogueira, Marcella Pecora Milazzotto

**Affiliations:** ^1^Center of Natural and Human Sciences, Universidade Federal do ABC, Santo André, SP, Brazil; ^2^Institute of Biomedical Sciences, Universidade de São Paulo, SP, Brazil; ^3^Institute of Biosciences, Campus Botucatu, Department of Pharmacology, Universidade Estadual Paulista (UNESP), Botucatu, SP, Brazil; ^4^School of Sciences and Languages, Campus Assis, Department of Biological Sciences, Universidade Estadual Paulista (UNESP), Assis, SP, Brazil

## Abstract

High oxygen levels during *in vitro* culture (IVC) can induce oxidative stress through accumulation of reactive oxygen species (ROS), negatively affecting embryo development. This study evaluated the effect of different O_2_ tensions during IVC on bovine blastocyst development and transcriptional status, considering transcription factors that play an essential role during early embryo development. For this purpose, embryos were produced *in vitro* by conventional protocols and cultured in two different oxygen tensions, physiological (5%) and atmospheric (20%). Expanded blastocysts were subjected to transcript quantitation analysis by RT-qPCR with Biomark™ HD System (Fluidigm, US), using 67 TaqMan assays specific for *Bos taurus*. Differences were observed in genes related to oxidation-reduction processes, DNA-dependent transcription factors, and factors related to important functional pathways for embryo development. Blastocyst rate was higher in the 5% O_2_ group and the number of cells was assessed, with the 5% O_2_ group having a higher number of cells. ROS concentration was evaluated, with a higher ROS presence in the 20% O_2_ group. Taken together, these results allow us to conclude that IVC of embryos at atmospheric O_2_ tension affects the expression of important transcription factors involved in multiple cell biology pathways that can affect embryo development, quality, and viability.

## 1. Introduction

The study of the embryo *in vitro* production (IVP) leads to a better knowledge of cellular and molecular aspects that occur and control the early embryonic development. Transcriptome analysis studies, with focus on genes related to embryo development and quality [1], stress response [2–4], metabolism [5], genetic loss, and embryo survival [6], demonstrate that the analysis of molecular parameters continue to be essential for the understanding of biochemical processes involved in development of healthy embryos.

During the IVP, *in vitro* culture (IVC) has a great impact on the quality of blastocysts. Essential development events occur during IVC, such as the first cleavages, whose kinetics interfere with the subsequent development of the embryo, genome activation, epigenetic reprogramming, morula compaction, and formation of the blastocyst, with the first morphological cellular differentiation via formation of the inner cell mass (ICM) and trophectoderm (TE) [7–9]. External influences and changes in the culture system can, therefore, affect these development events, causing changes in the transcription pattern of the embryo [3, 7, 10].

Oxidative homeostasis is considered one of the most fundamental factors that can affect the quality of an IVP embryo. The oxygen tension in the *in vivo* environment is much lower than the atmospheric air tension, corresponding to 5–7% in the mammalian oviduct while the atmospheric tension is around 20% [10, 11]. *In vivo* embryo development occurs in the lower oxygen tension of the oviduct and relies on factors that maintain the balance between the production of reactive oxygen species (ROS) and antioxidant enzymes, due to an efficient reduction-oxidation reaction (redox) system, which can guarantee an ideal environment for embryonic development [12, 13]. During IVC, embryos cannot rely on the protection of the enzymatic or nonenzymatic antioxidants present in the physiological environment and there are studies that show that the culture media, depending on the composition, can be itself a source of ROS [14]. Therefore, IVC of embryos in high atmospheric oxygen tension can lead to ROS accumulation and induce oxidative stress in embryo cells without the enzymatic protection of antioxidants [10, 11, 13, 15].

Although a physiological concentration of ROS is necessary to maintain normal embryo development, studies have demonstrated the harmful effects of ROS accumulation on development of IVP embryos of several different species (mouse, hamster, rabbit, swine, sheep, and cattle), due to its interference with important cellular processes [10, 12, 15, 16]. In these studies, ROS damage affects DNA, proteins, lipids, and cellular structures, such as membranes, mitochondria, and endoplasmic reticulum [4, 10, 13, 17–20]. ROS damage to key cell components can cause changes in the expression of transcription factors and genes; DNA fragmentation and alteration in methylation pattern; interference in pluripotency and cell differentiation states; increased incidence of apoptosis; reduction in the number of embryo cells (ICM and TE); metabolic changes, such as depletion of ATP levels; change in ion channels; deleterious effects on protein synthesis; lipid peroxidation and alterations in membrane permeability; alterations on mitochondrial and endoplasmic reticulum functions; and embryo fragmentation and blocks [4, 10, 12, 15, 16, 21, 22].

The study of the oxidative stress impact on embryonic cells, especially resulting in changes in transcription factors that play an essential role in controlling the transcriptional profile during early embryo development, is important for a better understanding of these factors' functions and pathways, as well as their relation with molecular mechanisms of gene regulation, such as epigenetics mechanisms, which can affect the development of healthy IVP embryos [13, 15, 23–25]. Considering the oxidative stress' effects on transcription factors that have important roles during early embryo development are still unclear, we evaluated the interference of different oxygen tensions during IVC on the transcriptional status of bovine embryos, especially considering DNA-dependent transcription factors related to development, stress, and embryo morphophysiological characteristics.

## 2. Materials and Methods

### 2.1. Experimental Design

Oocytes were obtained from bovine ovaries from a commercial slaughterhouse. Oocytes were *in vitro* matured (IVM) and fertilized (IVF) following conventional protocols [26]. At the start of IVC, presumptive zygotes were cultured in two different oxygen tensions: atmospheric (20%) and near physiological (5%). Embryos of both groups were cultured at different O_2_ tensions until day 9 (D9) postinsemination for morphological and transcript pattern evaluations. Cleavage rate was recorded at 52 h postinsemination (hpi). At 186 hpi (D7), expanded blastocysts (XB) were collected for subsequent RT-qPCR using Biomark HD Real-Time PCR system (Fluidigm, USA) and morphological analysis (ROS detection and differential staining for cell counting). The blastocyst rate was calculated considering embryos that progressed to blastocyst stages in the period from D7 to D9.

### 2.2. *In Vitro* Embryo Production (IVP)

Bovine ovaries were obtained from a commercial slaughterhouse and cumulus-oocyte complexes (COCs) were collected by aspiration of follicles with 2–8 mm in diameter. Grade I and II COCs were selected according to cytoplasm aspect and number of layers of cumulus cells for *in vitro* maturation (IVM). In this study, a total of 1475 oocytes were used in 3 replicates. Groups of 25–30 COCs were placed in 90 *μ*L droplets of IVM medium [TCM-199 bicarbonate supplemented with 10% fetal bovine serum (FBS), 0.5 *μ*g/mL FSH (Folltropin-V, Bioniche, Canada), 100 IU/mL hCG (Chorulon, Merck Animal Health, Netherlands), 0.2 mM Na pyruvate (Sigma, US), 50 *μ*g/mL gentamicin sulfate (Sigma, US), and 1.0 *μ*g/mL estradiol] under mineral oil for 24 h at 38.5°C and 5% CO_2_ with high humidity.

For *in vitro* fertilization (IVF), groups of 25–30 IVM oocytes were inseminated with 1 × 10^6^ Percoll-purified spermatozoa/mL from a single bull following conventional protocol [26] and incubated at 38.5°C and 5% CO_2_ with high humidity. After 18 h from the start of fertilization, remaining granulosa cells were removed and groups of 25–30 zygotes were transferred to 90 *μ*L droplets of synthetic oviduct fluid (SOF) culture medium supplemented with 2% essential amino acids (M-5550, Sigma, US), 1% nonessential amino acids (M-7145, Sigma, US), and 5% FBS (SOFaa). At this moment, the embryos were separated into two groups for culture under controlled atmosphere with different oxygen tensions. The group maintained in high oxygen tension (20%), denominated 20% O_2_ group, was cultured in the incubator at 38.5°C and 5% CO_2_ in atmospheric air and high humidity. The group cultured in physiological oxygen tension (5%), denominated 5% O_2_ group, was kept in small-sealed chambers with a gas mixture of 5% O_2_, 5% CO_2_ and 90% N_2_, and high humidity in an incubator at 38.5°C.

### 2.3. Reactive Oxygen Species (ROS) Detection

ROS detection in the embryo cells was done at the expanded blastocyst stage with the use of 2′,7′-dichlorofluorescein (DCF) probe (Thermo Fisher Scientific, USA). Probe detection, indicating ROS presence was made measuring the fluorescence intensity using the epifluorescence microscope Leica Microsystems DM16000 B (Leica Microsystems, Brazil). Fluorescence detection was made with the L5 filter (excitation/emission 495-519 nm). The protocol validated and used was an adaptation of Pontes et al. [27], for detection of ROS in bovine embryos.

Embryos were individually evaluated and photographed in the epifluorescence microscope. After obtaining the photos, XB were submitted to Hoechst 33342 staining, fixed with paraformaldehyde solution, placed on a slide, and photographed again for localization of the embryonic cells (blue filter I3—excitation/emission 350–490 nm). The probe and staining were made in ten XB of each group (20% O_2_ and 5% O_2_), and the three replicates and embryos were photographed at 40x magnification. Photos of each embryo were analyzed using ImageJ software (National Institutes of Health, USA). The fluorescence area was selected using the threshold tool, with the same threshold value (*t* = 30) being applied to all embryo images of the two groups. The final fluorescence intensity value for each embryo was measured using the mean gray values of the area selected by the threshold (MGT), the average of the mean gray values of 4 selected backgrounds (MB), and the total area of the embryo (TA). The following formula was applied to the values obtained from each embryo: fluorescence intensity = (MGT − MB)/TA. The statistical analysis was done using the average of the fluorescence intensity of the 10 XBs individually analyzed per group per replicate (*n* = 3 per group).

### 2.4. Transcript Quantitation by RT-qPCR

Expanded blastocysts (XB) from 20% O_2_ and 5% O_2_ groups were collected after 186 hpi (D7) and kept at −80°C until analysis. Each sample, consisted of a pool of 10 XB per sample from each group, were collected in 3 different replicates (*n* = 3 samples per group). Samples were processed for RNA extraction, cDNA synthesis, and submitted to RT-qPCR using Biomark HD Real-Time PCR (Fluidigm, USA).

Total RNA extraction was made with a PicoPure® RNA Isolation Kit (Applied Biosystems™, USA), and samples were stored at −20°C for subsequent cDNA synthesis. Total RNA was quantified with NanoDrop (NanoDrop™, Thermo Fisher Scientific, USA). Each sample (50 ng) were submitted to reverse transcriptase using the High-Capacity cDNA Reverse Transcription Kit (Applied Biosystems, USA), according to manufacturer's instructions, to a final reaction volume of 20 *μ*L.

Transcript quantitation analysis of the embryos was performed using TaqMan assays (Applied Biosystems, USA), specific for *Bos taurus* species. Prior to qPCR thermal cycling, each sample was submitted to sequence-specific preamplification process as follows: 1.25 *μ*L assay mix (TaqMan assay was pooled to a final concentration of 0.2X for each assay used), 2.5 *μ*L TaqMan PreAmp Master Mix (Applied Biosystems, USA), and 1.25 *μ*L cDNA. The reactions were activated at 95°C for 10 min followed by 14 cycles of denaturing at 95°C for 15 s and annealing and amplification at 60°C for 4 min. These preamplified products were diluted 6-fold prior to RT-qPCR analysis.

Each sample solution prepared for qPCR thermal cycling consisted of 2.25 *μ*L diluted-preamplified cDNA, 2.5 *μ*L of TaqMan Universal PCR Master Mix (2X, Applied Biosystems, USA), and 0.25 *μ*L of 20X GE Sample Loading Reagent (Fluidigm, USA). The assay solution consisted of 2.5 *μ*L of 20X TaqMan Gene Expression Assay (Applied Biosystems, USA) and 2.5 *μ*L of 2X Assay Loading Reagent (Fluidigm, USA). A 96.96 Dynamic Array™ Integrated Fluidic Circuits (Fluidigm, USA) chip was used for data collection. After priming, the chip was loaded with 5 *μ*L of each assay solution and 5 *μ*L of each sample solution. The qPCR thermal cycling was performed in the Biomark HD System (Fluidigm, USA) using the TaqMan GE 96 × 96 standard protocol, consisting of Thermal Mix (50°C for 2 min, 70°C for 20 min, and 25°C for 10 min), followed by a Hot start stage (50°C for 2 min and 95°C for 10 min), followed by 40 cycles of denaturation (95°C for 15 s) and extension (60°C for 60 s).

TaqMan assays used in this study (Supplementary Material—Table I available online at https://doi.org/10.1155/2017/1502489) were selected considering the analysis of DNA-dependent transcription factors related mainly to embryo development, oxidative stress, pluripotency and cell differentiation, cell proliferation, and apoptosis. PCR results with Ct values of all genes analyzed for the respective samples were obtained by the Biomark software (Biomark Real-time PCR Analysis, Fluidigm, USA). Housekeeping genes were selected analyzing most stable genes in all samples with NormFinder [28]. Housekeeping genes selected were beta actin (*ACTB*; Gene ID: BT.14186), glyceraldehyde-3-phosphate dehydrogenase (*GAPDH*; Gene ID: BT.87389), and peptidylprolyl isomerase A (*PPIA*; Gene ID: BT.43626). Relative expression values of each gene were calculated applying the ΔΔCt fold change method with the R package (Bioconductor) ddCt Algorithm for the Analysis of Quantitative Real-Time PCR 1.30.0 [29], using a control sample consisting of a pool of all samples as calibrator.

### 2.5. Morphological Analysis—Differential Staining of ICM and TE Cells

Analysis of the total number of cells, inner cell mass (ICM) cells, and trophectoderm (TE) cells was made in expanded blastocysts of the two studied groups in 3 replicates. Embryos were submitted to differential staining protocol adapted from Block et al. and Selokar et al. after validation tests [30–32]. Stained blastocysts were individually evaluated and photographed on the same day, at 40x magnification and different focal planes using the Leica Application Suite (LAS) of the Leica Microsystems DM16000 B epifluorescence microscope (Leica Microsystems Brazil).

Staining of the embryo TE cell nuclei with propidium iodide was visualized using the Y3 red filter (excitation/emission 538–617 nm), while total cells, nuclei stained by Hoechst 33342, were visualized with the I3 blue filter (excitation/emission 350–490 nm). Embryo photographs were processed in the Adobe Photoshop CS2 graphic software (Adobe Systems, USA) for the overlap of the layers of the different focal planes obtained, using the opacity tool. Visualization of ICM and TE cells was obtained with the use of transparency between the layers stained with Hoechst 33342 and propidium iodide, respectively. The statistical analysis was done considering the average of the number of cells of the 10 XBs individually stained per group per replicate (*n* = 3 per group).

### 2.6. Statistical Analysis

Embryo production data, cleavage, and expanded blastocysts rate were calculated via the total number of initial oocytes and were submitted to the D'Agostino-Pearson normality test and *t*-test in 3 replicates. In the analysis of gene expression variation, ΔΔCt fold change values of the samples (*n* = 3 per group) were submitted to *t*-test. Genes with *P* < 0.05 were considered as significantly different in relative expression between the analyzed groups (the *P* values for all genes are available in the Supplementary Material—Table III). The graphical representation of genes with significant difference between the two groups (20% O_2_ and 5% O_2_) was made using the log base 2 of the ΔΔCt fold change values, with the *x*-axis representing the control sample. Genes were separated into different functional pathways after being submitted to the Functional Annotation Tool (DAVID Bioinformatics Resources 6.7) [33]. Some genes are represented in more than one pathway due to its function overlay. For the morphological analysis data, ROS detection, and cell counting by differential staining, the D'Agostino-Pearson normalization test and *t*-test were done considering the average of the number of cells and the average of fluorescence intensity of the XBs individually analyzed per group per replicate (*n* = 3 per group). All analyses were performed using SAS System for Windows (SAS Institute Inc., USA) and Prism 5 GraphPad software (GraphPad Software Inc., USA).

## 3. Results

### 3.1. Oxidative Stress Negatively Influence Embryo Development

**Figure 1:**
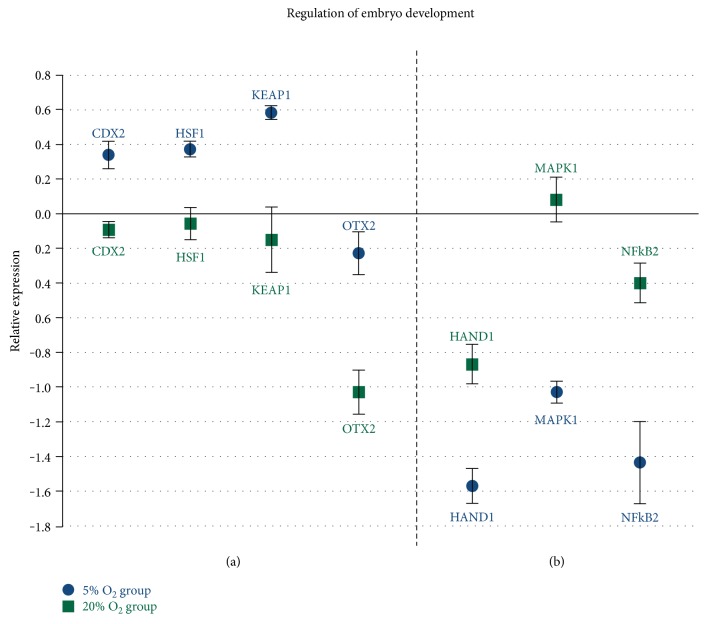
Genes related to embryo development regulation: (a) genes upregulated in the 5% O_2_ group (*P* < 0.05); (b) genes downregulated in the 5% O_2_ (*P* < 0.05); and *x*-axis (0) represents the control sample.

There was no difference in the cleavage rates between the 20% O_2_ and 5% O_2_ groups, but the 5% O_2_ group had a higher conversion rate of blastocysts (Table 1). There were also differences between groups in the relative abundance of transcripts related to the regulation of embryo development with *CDX2*, *HSF1*, *KEAP1*, and *OTX2* upregulated in the 5% O_2_ group (Figure 1(a)) and HAND1, MAPK1, and NFkB2 downregulated in the same group in comparison with the 20% O_2_ group (Figure 1(b)).

### 3.2. Oxidative Stress Influence the Transcription Pattern of REDOX and Cell Stress-Related Genes

**Figure 2:**
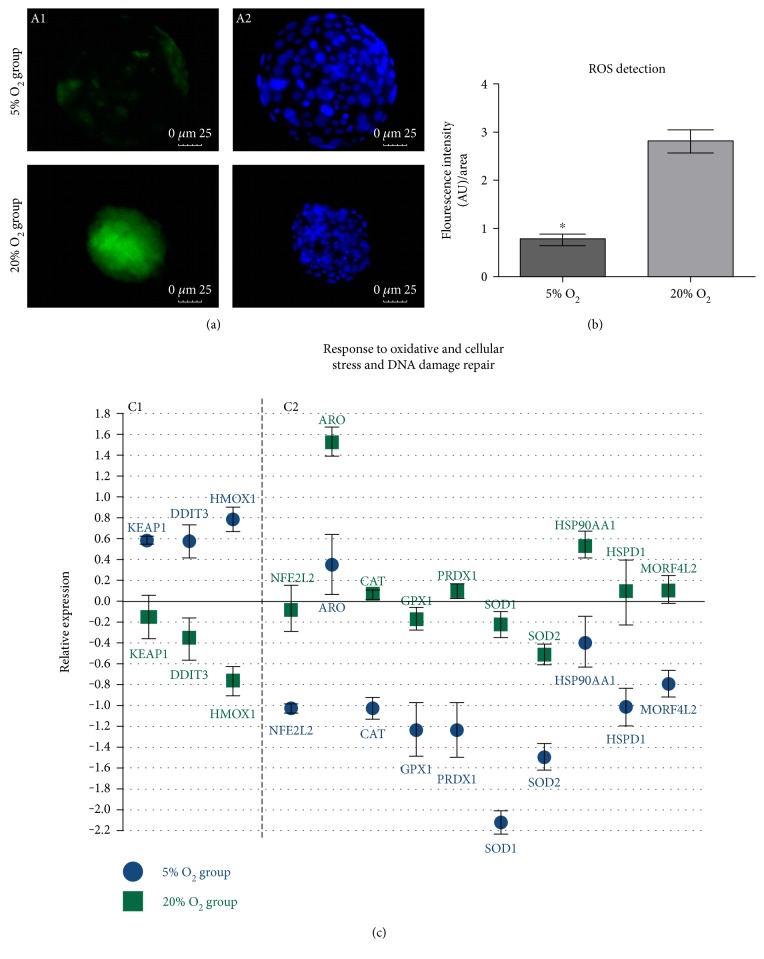
(a) DCF probe detection in expanded blastocysts from the 5% O_2_ and 20% O_2_ groups (40x magnification); A1—fluorescence intensity of DCF in the presence of ROS; A2—Hoechst 33342 staining of the embryo cells nuclei. (b) Difference of fluorescence intensity generated by DCF probe between the analyzed groups. Mean ± SEM of the groups 5% O_2_ (0.76 ± 0.15) and 20% O_2_ (2.80 ± 0.18) (*P* = 0.001). (c) Genes related to oxidative and cellular stress response and DNA damage repair with difference between the analyzed groups; C1—genes upregulated in the 5% O_2_ group (*P* < 0.05); C2—genes downregulated in the 5% O_2_ (*P* < 0.05); and *x*-axis (0) represents the control sample.

ROS detection through the evaluation of the fluorescence intensity generated by DCF probe, (Figure 2 A1), resulted in difference (*P* = 0.001) between the studied groups (Figure 2(b)). Embryos cultured in 5% oxygen tension presented a lower level of ROS (fluorescence intensity: 0.76 ± 0.15) in the 3 replicates analyzed in comparison with the group cultured at atmospheric oxygen tension (fluorescence intensity: 2.80 ± 0.18). Embryo response to oxidative stress was also observed in the difference of the transcription pattern between the groups (Figure 2(c)). There was a higher relative abundance of *KEAP1*, *DDIT3*, and *HMOX1* in the 5% O_2_ group, while the antioxidant response elements *NFE2L2*, *ARO*, *CAT*, *GXP1*, *PRDX1*, *SOD1*, and *SOD2* were upregulated in the 20% O_2_ group. Embryos cultured at atmospheric oxygen tension (20% O_2_) also presented higher relative abundance of *HSP90AA1*, *HSPD1*, and *MORF4L2* genes related to response to cellular stress and DNA damage repair.

### 3.3. Oxidative Stress Alters Morphological Characteristics of Embryos

**Figure 3:**
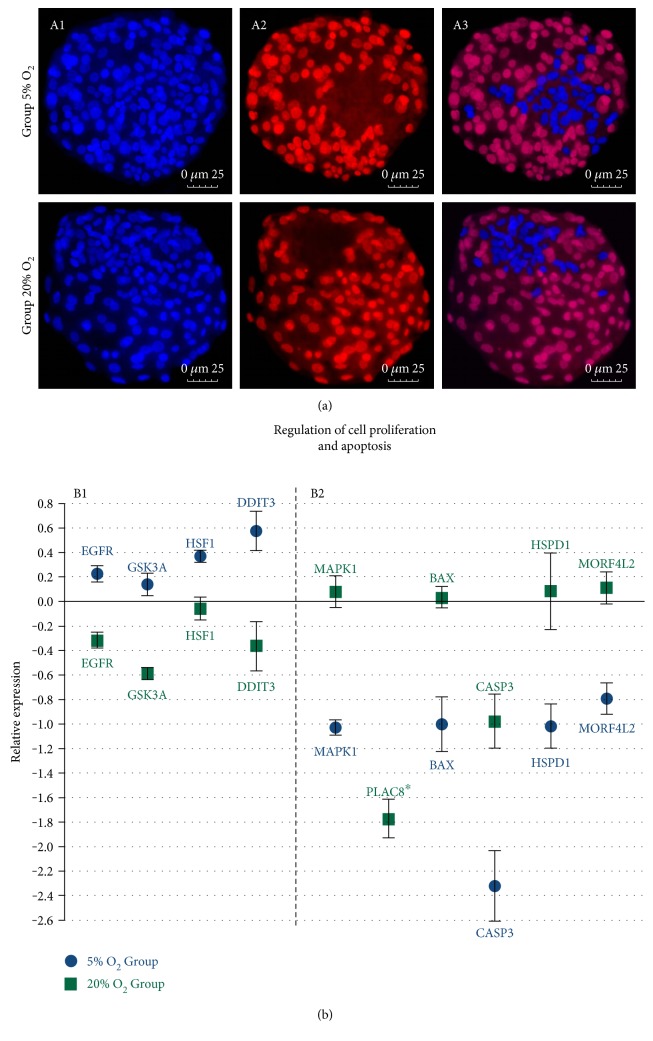
(a) Differential staining for cell counting in expanded blastocysts from the 5% O_2_ and 20% O_2_ groups (40x magnification); A1—total number of cells with nuclei marked by Hoechst 33342 staining; A2—TE cells nuclei marked by propidium iodide; and A3—ICM cells (blue) and TE cells visualized by overlapping the layers stained with Hoechst 33342 and propidium iodide. (b) Genes related to cell proliferation rate and apoptosis with difference between the analyzed groups; B1—genes upregulated in the 5% O_2_ group (*P* < 0.05); B2—genes downregulated in the 5% O_2_ (*P* < 0.05); and *x*-axis (0) represents the control sample. ^∗^detected only in the 20% O_2_ group.

Embryos cultured in different oxygen tension presented a difference in total, ICM, and TE cell numbers in the analysis of differential staining (Figure 3(a)). Embryos produced at 5% oxygen tension had a higher number of total, ICM, and TE cells (Table 2) in the 3 replicates in comparison with the atmospheric oxygen tension group.

There were also differences between the groups regarding genes related to cell proliferation, with higher relative abundance in the 5% O_2_ group of *HSF1*, *EGFR*, and *GSK3A*, while *MAPK1* was downregulated in this group and *PLAC8* was only detected in the 20% O_2_ group. In the analysis of apoptosis-related genes, which also influences the number of cells, the results show that the 5% O_2_ group had a lower relative abundance of *CASP3*, *HSPD1*, *BAX*, *MORF4L2*, and *PLAC8* and higher relative abundance of *DDIT3* transcripts.

### 3.4. Oxygen Tension Alters the Expression of Important Transcription Factors Related to Embryo Development

**Figure 4:**
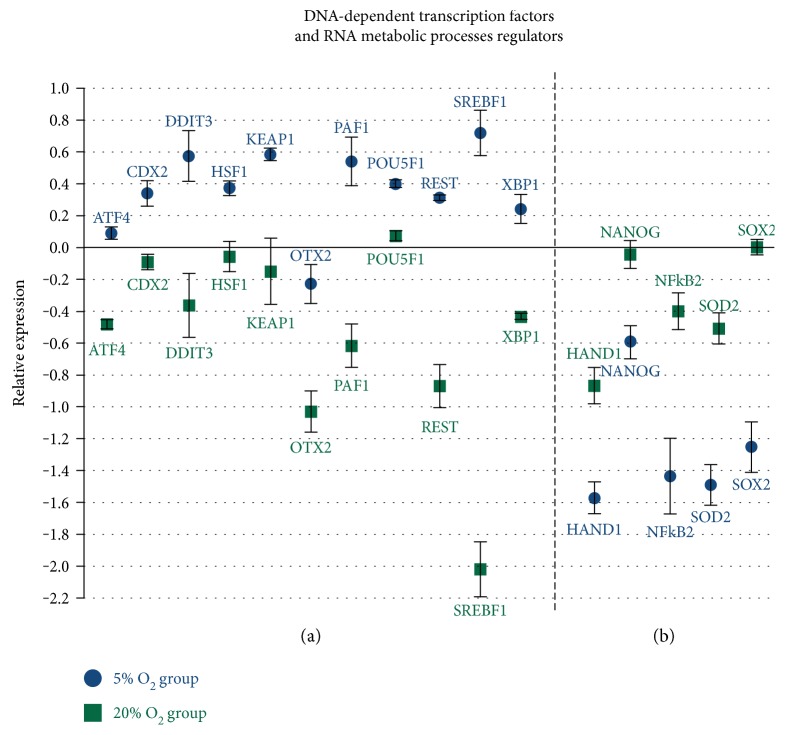
DNA-dependent transcription factors and RNA metabolic process regulators with difference between the analyzed groups. (a) Genes upregulated in the 5% O_2_ group (*P* < 0.05); (b) genes downregulated in the 5% O_2_ (*P* < 0.05); and *x*-axis (0) represents the control sample.

**Figure 5:**
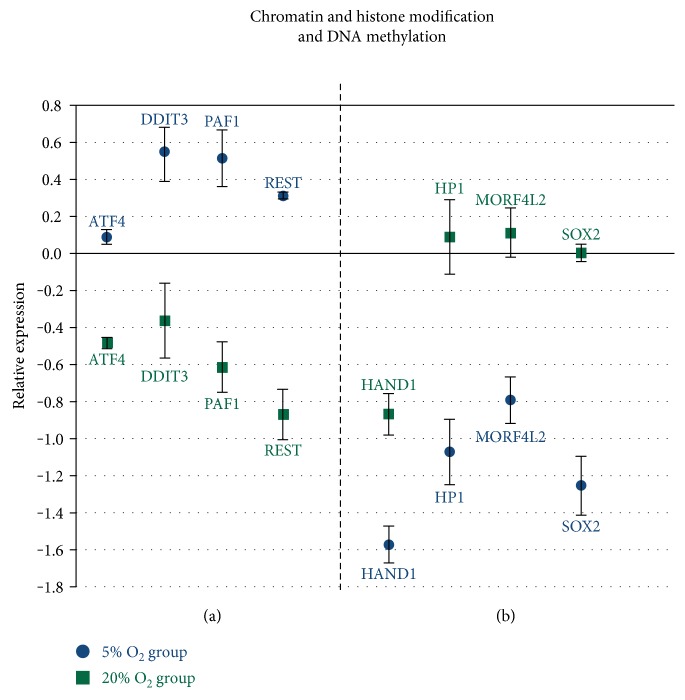
Genes related to chromatin and histone modification and DNA methylation with difference between the analyzed groups. (a) Genes upregulated in the 5% O_2_ group (*P* < 0.05); (b) genes downregulated in the 5% O_2_ (*P* < 0.05); and x-axis (0) represents the control sample.

**Figure 6:**
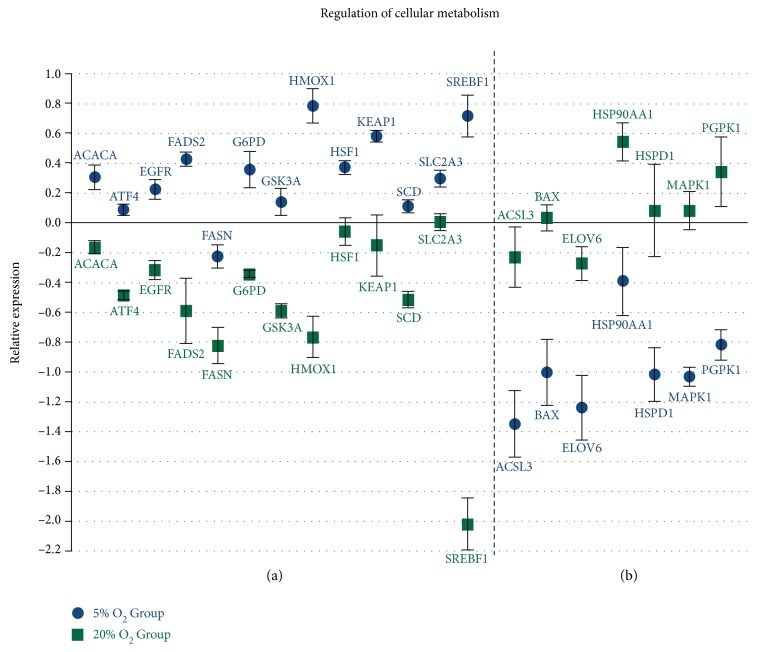
Genes related to regulation of cell metabolism with difference between the analyzed groups. (a) Genes upregulated in the 5% O_2_ group (*P* < 0.05); (b) genes downregulated in the 5% O_2_ (*P* < 0.05); and x-axis (0) represents the control sample.

The results observed in the present study show differences between the analyzed groups regarding transcription factors with functions in several distinct functional pathways that can impact embryo development. There was a difference in 16 DNA-dependent transcription factors with positive and negative regulation of gene expression, and that also are related to the regulation of RNA metabolic processes (Table 3 and Figure 4). Results of the transcription analysis also showed differences in factors related to maintenance of pluripotency state and cell differentiation (Figure 4) and epigenetic mechanisms such as chromatin and histone modification and DNA methylation (Figure 5). There was also a difference in the transcripts of 20 transcription factors and genes related to regulation of cell metabolisms, such as lipid biosynthesis, sugar metabolism, and protein folding processes and metabolism (Figure 6).

## 4. Discussion

Embryo production at oxygen tension near to the physiological (5–7% of O_2_) results in better development, with increased cleavage and blastocyst rates [10, 17, 21, 34]. In corroboration with these studies, embryos cultured at 5% oxygen tension in all replicates of this study had a higher blastocyst conversion rate than those cultured at atmospheric oxygen tension. However, no difference was observed in the cleavage rate of the embryos of the analyzed groups, which was also reported by Amin et al., 2014.

**Figure 7:**
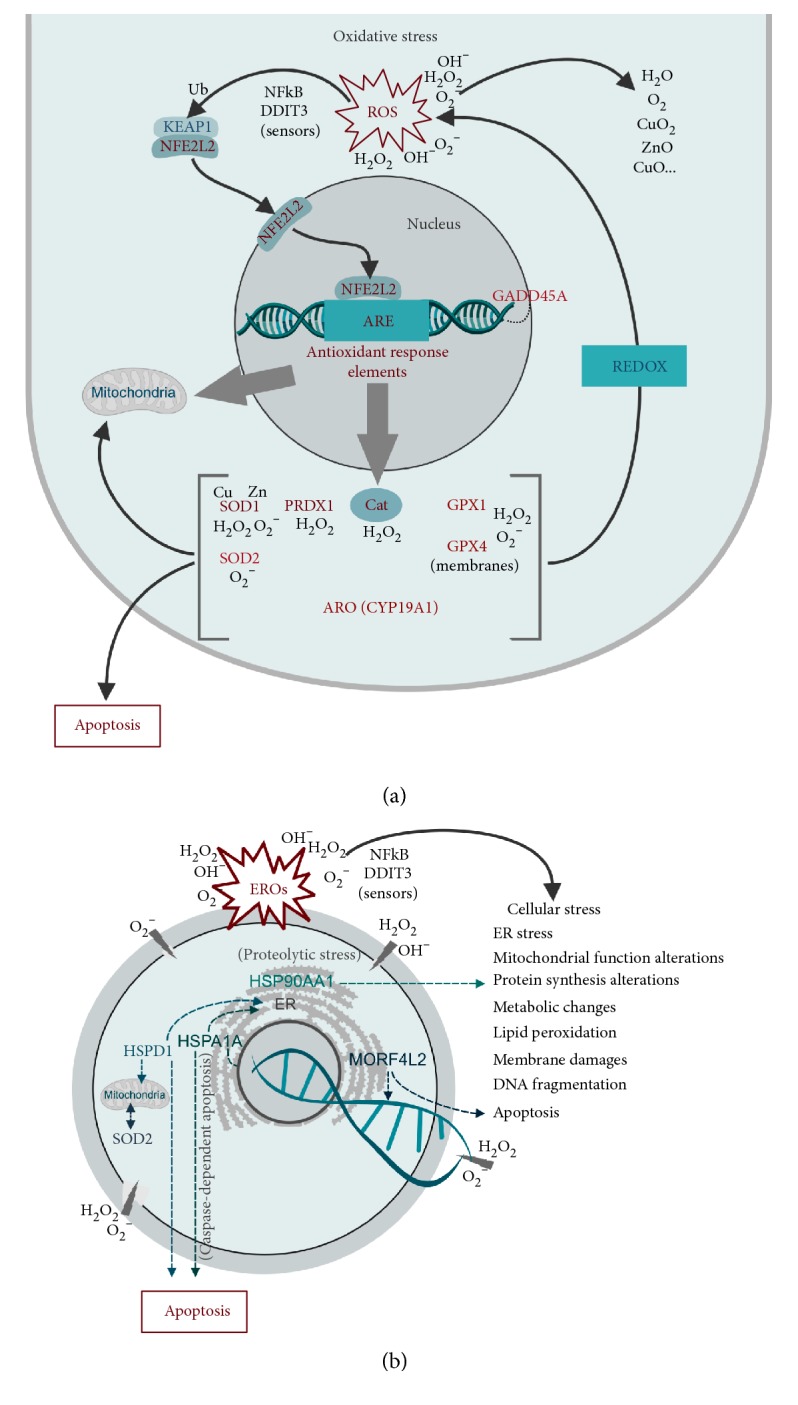
(a) Response to oxidative stress pathway controlled by *KEAP1*/*NFE2L2* interactions and antioxidant response elements triggered by *NFE2L2* (figure adapted from [10, 33]); (b) response to a broader cellular stress caused by oxidative stress (figure adapted from [21, 33]).

Higher oxygen levels (20% O_2_) during embryo *in vitro* culture also lead to an increase in ROS accumulation, which can promote many detrimental effects as observed in the difference of the transcription pattern of stress-related genes. The *NFE2L2*-mediated oxidative stress response pathway was selected in the present study, since *NFE2L2* binds to DNA-specific promoter regions of antioxidant response elements, triggering the expression of genes to act in the REDOX process. This pathway (Figure 7(a)), relating the functions of *NFE2L2* and *KEAP1* (*NFE2L2* inhibitory factor, keeping it sequestered to the cytosol), is considered dominant in the cellular oxidative stress response [10]. The results obtained in this study corroborate with those observed by Amin et al, 2014, with a higher expression of *NFE2L2* in the 20% O_2_ group and a higher expression of *KEAP1* in the 5% O_2_ group.

The response to oxidative stress analysis has to also consider genes activated by the *NFE2L2* factor pathway, which encodes antioxidant enzymes. As expected and in accordance with several studies [10, 13, 33, 35, 36], there was a higher abundance of transcripts of *CAT*, *SOD1*, *SOD2*, *PRDX1*, *GPX1*, and *ARO* in the 20% O_2_ group. The 5% O_2_ group presented more transcripts of *HMOX1* and *DDIT3*; however, these gene functions are not only related to cell protection against oxidative damage and cell stress. They can interact with other molecules activating other functional pathways related to cellular metabolism (*HMOX1*) and DNA target methylation (*DDIT3*), for instance. Therefore, considering the *NFE2L2*/*KEAP1* pathway and the activation of genes that works as antioxidant response elements, the group exposed to the atmospheric high oxygen tension showed a higher abundance of transcripts of genes related to oxidative stress, which may indicate the need for a REDOX response due to the ROS accumulation observed in their embryonic cells.

Oxidative stress can lead to a broader cell stress scenario, affecting mitochondrial and endoplasmic reticulum functions, causing metabolic alterations and DNA fragmentation, among other detrimental effects [4, 12, 16, 19, 21, 37]. In fact, a higher level of transcripts related to cell stress response and DNA damage repair, such as *HSP90AA1*, *HSPD1*, *MORF4L2*, and *SOD2*, was evidenced in embryos of the 20% O_2_ group (Figure 7(b)).

Results from oxidative stress studies also show differences in cell proliferation, with a significant increase in the total number of total cells and reduction in the proportion of apoptotic cells in embryos cultured in 5% oxygen tension, which can directly interfere in the number of ICM and TE cells of these embryos [10, 21, 38, 39]. In this study, embryos of the 5% O_2_ group had a higher total number of cells and also a higher number of TE and ICM cells. Thus, considering the morphological analysis of the number of embryo cells, IVC in near-physiological oxygen tension shows a positive influence on cell proliferation.

There were also differences between the groups regarding the transcripts of genes related to embryonic development and cell proliferation, with higher levels in the 5% O_2_ group of transcripts of *CDX2*, *HSF1*, *KEAP1*, *OTX2, EGFR*, and *GSK3A*, the last two directly related to cell proliferation stimulus. In the analysis of apoptosis-related genes, which also influences the number of cells and early embryo development, the 5% O_2_ group had a lower abundance of transcripts of apoptosis regulatory genes such as *CASP3*, *HSPD1*, *BAX*, *MORF4L2*, and *PLAC8*. Although the 5% O_2_ group had higher *DDIT3* transcripts, it is important to note that this factor has functioned in many distinct pathways and act in both positive and negative regulation of the programmed cell death process [33, 35, 40].

The results observed in the present study show differences between the analyzed groups regarding several transcription factors that have a great impact on embryo development. Taking into consideration DNA-dependent transcription factors, which act in processes that regulate the frequency, rate, and extent of DNA transcription, we observed a difference in the expression of 16 factors. In the 5% O_2_ group, there was a higher abundance of *ATF4*, *CDX2*, *DDIT3*, *KEAP1*, *HSF1*, *OTX2*, *PAF1*, *POU5F1* (*OCT4*), *REST*, *SREBF1*, and XBP1 factors that are related to several cell cycle processes. *CDX2* and *POU5F1*, for instance, participate in pathways related to pre- and postimplantation development, TE cells differentiation, and pluripotency regulation and are also involved with macromolecules biosynthesis processes and metabolic processes of nucleosides, nucleotides, and nucleic acids [33, 35, 41]. *DDIT3* interferes in multiple pathways by the positive regulation of gene expression and, as *ATF4*, plays a key role in DNA-specific target methylation by interacting with de novo methyltransferases [42, 43]. *PAF1* factor, due to its role in chromatin organization and histone modifications, regulates gene expression and pluripotency state [33, 35, 44]. *KEAP1*, *HSF1*, *OTX2*, *SREBF1*, and *XBP1* have functions on distinct metabolic pathways, macromolecules biosynthesis processes, and regulate the transcription of genes related to early embryonic development [10, 21, 33, 45].

The examples cited above indicate that the difference in the expression of these factors observed between the studied groups may interfere with embryonic development and their downregulation in embryos cultured in higher oxygen tension may result in damage, reversible or not, to the embryo cells [4]. An *XBP1* knockdown study with bovine embryos, for example, showed an elevation of ROS accumulation and reduction of antioxidant enzymes transcripts, affecting the development of the embryos. *XBP1* knockdown in mice results in a blockage of several cellular processes, interfering in proliferation, cell differentiation, and consequently in embryo survival [46].

DNA-dependent transcription factors downregulated in the 5% O2 group were *HAND1*, *NANOG*, *NFKB2*, *SOX2*, and *SOD2*. These factors also have important functions on several pathways; however, it is important to note that *HAND1*, *NANOG*, and *SOX2* negative regulate gene expression, and macromolecule metabolism and biosynthesis processes, while *SOD2* and *NFKB2* are important stress response factors [10, 33, 35, 47, 48].

Most of the transcription factors that presented differences between the studied groups (*ATF4*, *CDX2*, *DDIT3*, *HSF1*, *OTX2*, *POU5F1*, XBP1, *HAND1*, *NANOG*, *NFKB2*, *SOX2*, and *SOD2*) also have important functions in RNA metabolic processes, regulating the frequency, rate, and extent of chemical reactions involving RNA [33, 35].

The analysis of transcription factors related to maintenance of pluripotency state and cell differentiation showed a difference in *POU5F1* (present in the ICM and TE), *CDX2*, *REST*, and *PAF1*, upregulated in the 5% O_2_ group. *NANOG* (restricted to ICM), *SOX2* (restricted to ICM), *CDH1*, *HAND1*, and *MAPK1* (functions on cell differentiation and TE development) were downregulated in the 5% O_2_ group. However, further investigation of pluripotency and cell differentiation pathways in cattle is required, considering that many studies conclude that still little is known about these mechanisms [48].

The correlation between the transcription factors analyzed and several pathways important for embryo development also showed differences in factors with functions in chromatin and histone modification and with indirect interference in DNA methylation. *REST*, *PAF1*, *DDIT3*, and *ATF4* were upregulated in the 5% O_2_ group, while *HP1* and *SOX2* were downregulated in the same group. *REST* and *PAF1*, both factors related to pluripotency state pathways, also have functions in chromatin and histone modification [33, 35]. *ATF4*, *DDIT3*, and *HAND1* transcription factors are crucial in targeting DNA methylation, binding to specific areas of DNA and interacting with Dnmt3A and Dnmt3B [42, 49].

The higher abundance of transcripts of *HP1* and *SOX2* in the 20% O_2_ group corroborates with a study that shows that there is greater global methylation in embryos exposed to oxidative stress [22]. HP1-encoding protein induces heterochromatin formation and interacts with histones and DNMTs with the main function of silencing gene transcription [33, 35, 44]. *SOX2* factor, ICM restricted in bovine embryos [48], is associated with changes in DNA methylation pattern, chromatin modification, and negative control of gene expression [11, 50]. These results, therefore, indicate that differences in the expression of transcription factors caused by oxidative stress may interfere with the complex pathways of epigenetic modifications during embryo development critical moments, such as genome activation and epigenetic reprogramming [20, 51, 52].

Considering the transcription factors and genes with functions in cell metabolism, such as lipid biosynthesis and sugar and protein metabolism, many were upregulated in the 5% O_2_ group (Figure 7 and Supplementary Material—Table II). This difference may indicate that the cells of embryos cultured at 5% oxygen tension have higher metabolic activity. A more detailed analysis of these factors and gene functional pathways might help to explain and justify the higher quality of these embryos, cited in several studies [8, 10, 11, 21, 53].

In conclusion, there are significant differences between embryos cultured in near-physiological and atmospheric oxygen tensions. Embryos cultured in atmospheric oxygen tension (20%) presented higher ROS accumulation, as well as a higher abundance of transcripts related to oxidative stress response. In contrast, it was evidenced that embryos cultured at 5% oxygen tension showed a better embryo development and a higher conversion rate to blastocysts. Results also showed the relation between transcription factors involved in cell proliferation and apoptosis with a higher number of embryonic cells. The differences observed in DNA-dependent transcription factors related to important functional pathways for early embryo development indicate that the oxidative stress interference in gene transcription may cause developmental failures and compromise the viability of embryos, especially at this stage, when the genome activation and epigenetic reprogramming occurs.

## Supplementary Material

Supplementary Material - Table I. Taqman assays used for gene expression evaluation by qRT-PCR. Supplementary Material - Table II. Genes with difference between the 5%O_2_ and 20%O_2_ groups separated by functional pathways. Supplementary Material - Table III. P value of the genes with relative difference between the 5%O_2_ and 20%O_2_ groups (in alphabetical order).

## Figures and Tables

**Table 1:** Embryo production data of the 5% O_2_ and 20% O_2_ groups.

Number of replicates: 3	Group 5% O_2_	Group 20% O_2_	*P*
Total number of oocytes	739	736	
Cleavage rate (mean ± SEM)	62.7 ± 4.1	57.4 ± 2.5	0.4453^∗^
Blastocyst rate (mean ± SEM) (total blastocyst/total oocytes—from D7 to D9)	38.7 ± 2.2	25.5 ± 1.1	0.0234
Blastocysts/cleaved rate (mean ± SEM) (from D7 to D9)	62.4 ± 3.6	44.7 ± 1.9	0.0366

^∗^No difference.

**Table 2:** Evaluation in the number of cells in expanded blastocysts of the 5% O_2_ and 20% O_2_ groups.

Number of replicates: 3	Group 5% O_2_	Group 20% O_2_	*P*
Total number of cells (mean ± SEM)	207.6 ± 2.9	179.7 ± 1.1	0.0009
TE cells (mean ± SEM)	150.3 ± 3.0	131.6 ± 1.8	0.0058
ICM cells (mean ± SEM)	57.4 ± 0.5	48.1 ± 0.7	0.0005

The statistical analysis was done using the average of the number of cells of the embryos individually analyzed (10 Bxs) per group per replicate (*n* = 3 replicates).

**Table 3:** Transcription factors with difference between the 5% O_2_ and 20% O_2_ groups separated by functional pathways.

Function	Transcription factors
DNA-dependent transcription factors (processes that regulate frequency, rate, and extent of DNA transcription)	*ATF4 CDX2 DDIT3 KEAP1 HSF1*
	*OTX2 PAF1 POU5F1*
	*REST SREBF1 XBP1*
	**HAND1 NANOG NFKB**2 **SOD2 SOX2**

Positive regulation of gene expression	*ATF4 DDIT3 KEAP1 SREBF1*

Negative regulation of gene expression	*REST*
	**HAND1 NANOG SOX2**

DNA-dependent transcription factors with specific binding	*DDIT3 ATF4 HSF1*
	*POU5F1* *OTX2*
	**NANOG NFKB2 SOX2**

RNA metabolic process regulators (processes that regulate frequency, rate, and extent of chemical reactions involving RNA	*ATF4 DDIT3 CDX2 HSF1*
	*POU5F1* *OTX2 XBP1*
	**HAND1 NANOG NFKB2 SOD2 SOX2**

Genes in italic upregulated in the 5% O_2_ group in comparison to the 20% O_2_ group. Genes in bold downregulated in the 5% O_2_ group in comparison to the 20% O_2_ group.
