# Corrigendum to “Oxidative Stress Alters the Profile of Transcription Factors Related to Early Development on *In Vitro* Produced Embryos”

**DOI:** 10.1155/2018/6730857

**Published:** 2018-03-20

**Authors:** Roberta Ferreira Leite, Kelly Annes, Jessica Ispada, Camila Bruna de Lima, Érika Cristina dos Santos, Patricia Kubo Fontes, Marcelo Fábio Gouveia Nogueira, Marcella Pecora Milazzotto

**Affiliations:** ^1^Center of Natural and Human Sciences, Universidade Federal do ABC, Santo André, SP, Brazil; ^2^Institute of Biomedical Sciences, Universidade de São Paulo, São Paulo, SP, Brazil; ^3^Institute of Biosciences, Campus Botucatu, Department of Pharmacology, Universidade Estadual Paulista (UNESP), Botucatu, SP, Brazil; ^4^School of Sciences and Languages, Campus Assis, Department of Biological Sciences, Universidade Estadual Paulista (UNESP), Assis, SP, Brazil

In the article titled “Oxidative Stress Alters the Profile of Transcription Factors Related to Early Development on *In Vitro* Produced Embryos” [1], there was a missing grant and the Acknowledgments section should be corrected as follows:

The authors are grateful to the institution UFABC and to the Brazilian Science Foundations' CNPq, CAPES, and FAPESP for their financial assistance and to Seleon Biotecnologia Animal Ltda. and Conselho Nacional da Pecuária de Corte (CNPC) for their support (Grant no. 2015/03381-0, São Paulo Research Foundation (FAPESP)).
