# Supplementary material for: Oxidative Stress Alters the Profile of Transcription Factors Related to Early Development on In Vitro Produced Embryos
Source: Oxid Med Cell Longev. 2017 Oct 25;2017:1502489. doi: 10.1155/2017/1502489 (PMC5676474; doi:10.1155/2017/1502489)
Supplement: Supplementary file 1 — Supplementary Material - Table I. Taqman assays used for gene expression evaluation by qRT-PCR. Supplementary Material - Table II. Genes with difference between the 5%O2 and 20%O2 groups separated by functional pathways. Supplementary Material - Table III. P value of the genes with relative difference between the 5%O2 and 20%O2 groups (in alphabetical order). [file 1502489.f1.pdf]

Supplementary Material - Table I – Taqman assays used for gene expression evaluation by qRT-PCR

Gene		ThermoFisher TaqMan code	Gene ID UniGene
ACTB	actin, beta	PA5-16914	BT.14186
GAPDH	glyceraldehyde-3-phosphate dehydrogenase	Bt03210912_g1	Bt.87389
PPIA	Peptidylprolyl Isomerase A	Bt03224617_g1	Bt.43626
NFE2L2	Nuclear factor (erythroid-derived 2)-like 2	Bt03251880_m1	BT.17324
KEAP1	Kelch-like ECH-associated protein 1	Bt03817661_m1	BT.2184
CAT	Catalase	Bt03228713_m1	BT.48925
SOD1	superoxide dismutase 1, soluble	Bt03215423_g1	BT.49637
SOD2	superoxide dismutase 2, mitochondrial	Bt03244551_m1	BT.4748
GPX1	Glutathione Peroxidase 1	Bt03259217_g1	BT.4317
GPX4	Glutathione peroxidase 4	Bt03259611_m1	BT.49234
PRDX1	Peroxiredoxin-1	Bt03223684_m1	BT.65324
HSPA1A	heat shock 70kDa protein 1A	Bt03292670_g1	Bt.49659
ARO (CYP19A1)	aromatase	Bt03213774_m1	Bt.4447
HSP90AA1	Heat Shock Protein 90kDa Alpha	Bt03218068_g1	Bt.61915
HSPA5	Heat Shock 70kDa Protein 5	Bt03244883_m1	Bt.65104
HSPD1	Heat shock 60kDa protein 1A	Bt04301477_g1	Bt.24270
ATF4	Activating transcription factor 4	Bt03221057_m1	BT.49442
CDX2	Caudal type homeobox 2	Bt03649157_m1	Bt.46244
DDIT3	DNA-damage-inducible transcript 3	Bt03251320_g1	BT.65257
NFKB2	Nuclear Factor Of Kappa Light Polypeptide Gene Enhancer In B-Cells 2	Bt03272789_g1	Bt.20288
HAND1	heart and neural crest derivatives expressed 1	Bt04318733_g1	Bt.46230
HSF1	Heat Shock Transcription Factor 1	Bt03249686_m1	Bt.61991
OTX2	Orthodenticle Homeobox 2	Bt04316301_g1	Bt.87811
PAF1	RNA Polymerase II Associated Factor	Bt03239371_g1	BT.13382
REST	RE1-silencing transcription factor	Bt03278318_s1	BT.86225
SREBF1	Sterol Regulatory Element Binding Transcription F1	Bt03276370_m1	Bt.15667
XBP1	X-Box Binding Protein 1	Bt03227621_g1	Bt.89117
DNMT1	DNA (Cytosine-5-)-Methyltransferase 1	Bt03224737_m1	BT.108052
DNMT3A	DNA (Cytosine-5-)-Methyltransferase 3A	Bt01027164_m1	BT.64560
DNMT3B	DNA (Cytosine-5-)-Methyltransferase 3B	Bt03259810_m1	BT.22977
HP1	Heterochromatin protein 1	Bt03246076_m1	BT.22333
NANOG	Nanog homeobox	Bt03220541_m1	BT.47449
POU5F1 (OCT4)	POU class 5 homeobox 1	Bt03223846_g1	BT.92603
SOX2	SRY (sex determining region Y)-box 2	Bt03278318_s1	BT.103364
H1F1A	Hypoxia inducible factor 1 (transcription factor)	Bt03259341_m1	BT.4184

Gene		ThermoFisher TaqMan code	Gene ID UniGene
MAPK1	Mitogen-Activated Protein Kinase	Bt03216718_g1	Bt.109487
EGFR	epidermal growth fator receptor	-	Bt.6422
IGF1R	Insulin-Like Growth Factor 1 Receptor	Bt03649217_m1	Bt.12759
IGFBP2	Insulin-Like Growth Factor Binding Protein 2	Bt01040719_m1	Bt.149
IGFBP4	Insulin-Like Growth Factor Binding Protein 4	Bt03259500_m1	Bt.5237
IFITM3	Interferon Signaling	Bt03292973_g1	Bt.32359
IFNT2	interferon tau	Bt03210579_g1	Bt.328
PLAC8	placenta-specific 8	Bt03211579_m1	Bt.64679
GSK3A	Glycogen Synthase Kinase 3a	Bt03273698_g1	Bt.33944
HMOX1	Heme oxygenase	Bt03218624_m1	Bt.4001
VEGFA	Vascular Endothelial Growth Factor A	Bt03213282_m1	Bt.4138
BAX	BCL2-Associated X Protein	Bt03211777_g1	Bt.109788
CASP9	Caspase 9, apoptosis-related cysteine peptidase	Bt04282453_m1	Bt.66332
CASP3	caspase 3, apoptosis-related cysteine peptidase	Bt03250954_g1	Bt.10084
CDH1	cadherin 1	Bt03210093_g1	Bt.64827
MORF4L2	mortality factor 4 like 2	Bt03270996_m1	Bt.26405
ACACA	Acetyl-CoA carboxylase	Bt03213360_m1	Bt.88312
ACSL3	Acyl-CoA Synthetase 3	Bt04282138_m1	Bt.89521
ACSL6	Acyl-CoA Synthetase 6	Bt03231692_m1	Bt.11904
ELOVL6	Fatty acid elongase 6	Bt00907566_m1	Bt.2073
FADS2	Fatty acid desaturase 2	Bt03256255_g1	Bt.3891
FASN	Fatty acid synthase	Bt03210471_g1	Bt.30099
SCD	Stearoyl-CoA desaturase	Bt04307477_m1	Bt.65021
AUH	AU RNA binding protein	Bt03275798_m1	Bt.92325
AQP3	aquaporin 3	Bt03253663_m1	Bt.53350
PFKP	Phosphofructokinase	Bt04316551_m1	Bt.3794
PGK1	Phosphoglycerate Kinase 1	Bt03225854_mH	Bt.37560
SLC2A1	Solute Carrier Family 2 Member 1	Bt03215313_m1	Bt.4646
SLC2A3	Solute Carrier Family 2 Member 3	Bt03259513_g1	Bt.3964
SLC2A5	Solute Carrier Family 2 Member 5	Bt03258299_g1	Bt.19805
AKR1B1	Aldo-Keto Reductase Family 1, Member B1	Bt03218049_g1	Bt.63116
G6PD	Glucose 6 phosphate dehydrogenase	Bt03649181_m1	Bt.65227

Assays used were separated into the following categories:

Housekeeping genes
Oxidative stress and response to cellular stress;
DNA-dependent transcription factors;
Related to epigenetic factors;
Related to pluripotency and cell differentiation;
Related to embryo development and cell proliferation;
Related to apoptosis;
Related to metabolism;

Supplementary Material - Table II – Genes with difference between the 5%O_2 and 20%O_2 groups separated by functional pathways

Functional Pathways	Genes
Redox process in response to oxidative stress	*KEAP1 DDIT3 HMOX1*<br>*NFE2L2 ARO (CYP19A1) CAT GXP1*<br>*PRDX1 SOD1 SOD2*
Response to cellular stress and DNA damage repair	*DDIT3*<br>*HSP90AA1 HSPD1 MORF4L2 SOD2*
DNA-dependent transcription factors (processes that regulate frequency, rate and extent of DNA transcription)	*ATF4 CDX2 DDIT3 KEAP1 HSF1*<br>*OTX2 PAF1 POU5F1 (OCT4)*<br>*REST SREBF1 XBP1*<br>*HAND1 NANOG NFKB2 SOD2 SOX2*
Positive regulation of gene expression	*ATF4 DDIT3 KEAP1 SREBF1*
Negative regulation of gene expression	*REST*<br>*HAND1 NANOG SOX2*
DNA-dependent transcription factors with specific binding	*DDIT3 ATF4 HSF1*<br>*POU5F1 (OCT4) OTX2*<br>*NANOG NFKB2 SOX2*
RNA metabolic process regulators (processes that regulate frequency, rate and extent of chemical reactions involving RNA	*ATF4 DDIT3 CDX2 HSF1*<br>*POU5F1 (OCT4) OTX2 XBP1*<br>*HAND1 NANOG NFKB2 SOD2 SOX2*
Chromatin and histone modification and DNA methylation	*ATF4 DDIT3 REST*<br>*HAND1 HP1 MORF4L2 SOX2*
Regulation of pluripotency	*CDX2 PAF1 POU5F1 (OCT4) REST*<br>*NANOG SOX2*
Regulation of cell differentiation	*CDX2*<br>*NANOG SOX2*
Regulation of TE differentiation process	*CDX2*<br>*HAND1 MAPK1*
Regulation of embryo development (blastocyst)	*CDX2 HSF1 KEAP1 OTX2*<br>*HAND1 MAPK1 NFKB2*
Regulation of cell proliferation rate	*EGFR GSK3A HSF1*<br>*MAPK1 PLAC8***
Regulation of apoptosis	*DDIT3*<br>*BAX CASP3 HSPD1 MORF4L2 PLAC8***
Regulation of cellular metabolism (sugars, amino acids, protein folding processes and protein activities regulation, fatty acid biosynthetic process)	*ACACA ATF4 EGFR FADS2 FASN*<br>*G6PD GSK3A HMOX1 HSF1*<br>*KEAP1 SCD SLC2A3 SREBF1*<br>*ACSL3 BAX ELOVL6 HSP90AA1*<br>*HSPD1 MAPK1 PGK1*

Genes in **blue**: up-regulated in the 5%O_2 group in comparison to the 20%O_2 group
Genes in **red**: down-regulated in the 5%O_2 group in comparison to the 20%O_2 group
(*) Detected only in the 20%O_2 group.

Supplementary Material - Table III – P value of the genes with relative difference between the 5%O_2 and 20%O_2 groups (in alphabetical order).

Gene	P value	Gene	P value
ACACA	0.011	***HSPD1***	0.0109
ACSL3	0.0244	***KEAP1***	0.014
ARO (CYP19A1)	0.0165	***MAPK1***	0.0044
ATF4	0.0004	***MORF4L2***	0.0107
BAX	0.0233	***NANOG***	0.0156
CASP3	0.0268	***NFE2L2***	0.0343
CAT	0.0004	***NFKB2***	0.0105
CDX2	0.0131	***OTX2***	0.0146
DDIT3	0.0232	***PAF1***	0.0076
EGFR	0.0049	***PGK1***	0.0319
ELOVL6	0.0127	***PLAC8****	< 0.0001
FADS2	0.0048	***POU5F1 (OCT4)***	0.003
FASN	0.0125	***PRDX1***	0.0023
G6PD	0.0106	***REST***	0.0002
GPX1	0.0128	***SCD***	0.0008
GSK3A	0.004	***SLC2A3***	0.0221
HAND1	0.0119	***SOD1***	0.001
HMOX1	0.0016	***SOD2***	0.0039
HP1	0.0243	***SOX2***	0.0005
HSF1	0.0116	***SREBF1***	0.0009
HSP90AA1	0.0225	***XBP1***	0.0042

(*) Detected only in the 20%O_2 group
